# Sensing the Snacking Experience: Bodily Sensations Linked to the Consumption of Healthy and Unhealthy Snack Foods—A Comparison between Body Mass Index Levels

**DOI:** 10.3390/foods13030438

**Published:** 2024-01-29

**Authors:** Chanette Frederiksen, Derek Victor Byrne, Barbara Vad Andersen

**Affiliations:** 1 Food Quality Perception and Society Team, iSense Lab, Department of Food Science, Faculty of Technical Sciences, Aarhus University, 8000 Aarhus, Denmark; chanette.frederiksen@food.au.dk (C.F.); derekv.byrne@food.au.dk (D.V.B.); 2 Sino-Danish College (SDC), University of Chinese Academy of Sciences, Beijing 101408, China

**Keywords:** interoception, food-related pleasure, snack food consumption, eating behaviour

## Abstract

The World Health Organisation (WHO) has highlighted the need for immediate action regarding the escalating global prevalence of overweight and obesity. Based on the need for long-term strategies supporting dietary behaviour changes, the concept of ‘interoception’ (i.e., sensing the body’s internal state) has been proposed as promising for understanding and controlling food intake behaviours. As eating behaviours are motivated by the need for securing energy demands as well as the desire for pleasure, investigating the bodily sensations perceived in relation to food consumption can support our understanding of human food and eating behaviours. Here, a consumer study was conducted on 286 Danish consumers to explore the interoceptive experience of snack food consumption. This study included an investigation of the consumers’ interoceptive capabilities and ability to feel pleasure, intuitive eating behaviours, snack food consumption frequency, the drivers of snack food consumption, food-related pleasure, and post-ingestive sensations linked to snack food consumption. The study was conducted on consumers with different Body Mass Index (BMI) levels to study potential differences between these groups. The study showed normal interoceptive capabilities and abilities to feel pleasure, with no differences between BMI groups. Regarding intuitive eating, the study found intuitive eating to be more prevalent among the group holding normal BMI. No significant differences between BMI groups were found in terms of snack intake frequency. However, differences between BMI groups were found in terms of: the drivers of snack consumption, the aspects bringing food-related pleasure from snack consumption, and the post-ingestive sensations felt after eating snack foods.

## 1. Introduction

The World Health Organization (WHO) emphasises the urgency of addressing the rising global rates of overweight and obesity [1]. With this request, it remains a necessity to create behavioural strategies that can effectively complement current efforts, as conventional approaches frequently prove insufficient for long-term success [2]. Interoception is defined as the sensing of the body’s internal state [3], and the concept of ‘interoception’ is regarded as a tool for promoting long-term behavioural changes due to its emphasis on heightened self-awareness and mindfulness regarding internal bodily signals, and not requesting strict caloric control when applied [4,5].

Interoceptive sensations traditionally include hunger, fullness and thirst sensations, and human perception of these sensations is believed to be essential for guiding food- and eating behaviours [6,7]. In terms of using interoception explicitly as a strategy to affect both quantitative and qualitative aspects of consumption, perceiving these sensations, as well as sensations of mental and physical appetite more broadly and in combination with energetic and wellbeing-oriented sensations [8,9], is believed to lead to the development of a deeper relation with and ability to react to bodily needs. As such, interoception facilitates the adoption of a more conscious and intuitive eating practice, with the potential for more long term sustained behavioural changes [10]. Interoceptive strategies stand in contrast to traditional strategies by relying on the assumption that bodily signalling will guide physiological needs and believing that human perception, trust in these perceptions, and ability to react in response to these sensations can drive a healthier lifestyle, preventing (and possibly treating) overweight and obesity [10].

Investigating and using interoception as a strategy to prevent and treat overweight and obesity requires an overview and understanding of the specific sensations possible to be perceived by individuals in various eating contexts. This comprehension is essential for the development of successful, sustained interoceptive behavioural strategies. Since eating behaviours are motivated not only by a need for securing bodily energy demands but indeed also by a desire for pleasure and reward, investigating pleasure-related sensations linked to food intake seems more imperative than ever, as the literature on hedonic response remains rather fragmented [11].

Previous research focusing on interoception per se has shown variations in inter-individual interoceptive capabilities, and that when interoception is impaired, it can cause altered eating behaviour both in terms of over- and under-consumption [12,13,14]. Impairments in interoceptive capabilities are seen among, but are not limited to, overweight and obese subjects and are believed to be associated with elevated Body Mass Index (BMI) levels [14,15,16,17]. Lower interoceptive capabilities have been linked to higher BMI levels [17], and a greater tendency to consume in the presence of preferred foods [18]. In contrast, individuals with better interoceptive capabilities tend to value hunger and satiety signs (i.e., the intuitive eating approach), exhibit lower BMI levels, have fewer body weight concerns, and fewer diet restrictions [19]. However, at this point, causality is unclear in terms of whether impairments in interoception contribute to or are a consequence of weight gain and obesity [17]. Also, the perception of pleasure sensations has been shown to differ among subjects. Specifically, it has been found that the sub-constructs of pleasure, namely ‘wanting’ and ‘liking’, differ between BMI levels, with overweight and obesity associated with greater ‘wanting’ (i.e., motivation/desire) for unhealthy foods, regardless of the experience of ‘liking’ from the orosensory experience of eating [20,21,22].

Unhealthy foods, especially the consumption of unhealthy snack foods, have been connected to the risk of overweight and obesity due to their contribution to positive energy balance [23,24]. In today’s world, snacking constitutes close to one-third of our daily energy intake, and a substantial proportion of these snacks is characterised by being energy-dense while lacking in essential nutrients [25]. While previous studies have investigated reasons for snacking [26,27], little is known regarding the internal bodily sensations felt when consuming snacks and how they relate to snack product intake. Investigating the interoceptive sensations linked to snack food consumption is believed to provide fundamental insights into the specific sensations felt in response to eating, facilitating a comparison of the sensations felt after consuming of different types of snacks and food in general and a comparison between different segments of consumers regarding their interoceptive capabilities. Longer-term, this clarification of interoceptive sensations can be used in studies that aim to investigate increased attention on internal sensations as a tool for changing eating behaviours.

The current study operates at the conscious level of interoception by focusing on consumers’ conscious sensing of bodily signals, including appetite and post-ingestive sensations, as well as pleasure-related sensations, in the context of healthy and unhealthy snack food consumption, with the overall aim of investigating consumers’ interoceptive experiences during snack food consumption in greater detail and elucidating potential differences between consumers from different BMI segments. More specifically, this study aimed to investigate consumers’ capabilities to feel pleasure, intuitive eating behaviours, snack food consumption frequency, the drivers of their snack food consumption, food-related pleasure, and post-ingestive sensations linked to their snack food consumption.

## 2. Materials and Methods

### 2.1. Study Design

The consumer study was conducted as an online self-report survey among 286 healthy Danish subjects with a BMI equal to 18.5 or above. The respondents completed the questionnaire in an at-home setting using any given electronic device. 

### 2.2. Respondents and Recruitment

Participants were recruited by sharing a link to the online questionnaire on the social platforms Facebook^®^, LinkedIn^®^, and Instagram^®^, and via health professionals (i.e., dietitians and clinical dietitians) who volunteered to share the questionnaire online. Accompanying the link was a text explaining the questionnaire’s purpose, outlining respondents’ rights, and emphasising voluntary and anonymous participation. This approach was taken to ensure transparency and to provide potential respondents with clear information before they decided to take part in the study. 

Inclusion criteria included being generally healthy, 18 years or older, having a BMI equal to 18.5 or above, being Danish-speaking, not suffering from allergies or sickness, not using medicine, not excluding foods from their diet, and not experiencing an abnormal appetite. Screening questions were provided at the beginning of the questionnaire to check if the inclusion criteria were met. If one or several of the inclusion criteria were not met, the respondents were guided out of the questionnaire and thanked for their interest (n = 131). For the criteria on BMI, participants were asked to provide their height and weight, and based on the calculation of BMI (weight (kg)/height (m)^2^), respondents with a BMI < 18.5 or those who failed to report their weight and height were excluded prior to data analyses (n = 11). A total of 142 respondents were excluded due to not meeting the inclusion criteria. The questionnaire was online from March 2023 to May 2023, and data were collected via the Compusense^®^ Cloud software (Compusense Inc., Version 23.0.27 Guelph, ON, Canada) [28]. In total, the data analysis was based on 286 respondents (240 females; 45 males; 1 whose gender was not reported) with a mean age of 37.94 years (SD = 14.54). 

Characteristics of the respondents can be seen in Table 1. All respondents gave written consent prior to commencing the questionnaire. Due to the Danish legislation on non-biological material data in research, the study did not require approval from the Central Denmark Region Committees on Health Research Ethics (Section 14 (2) in the Committee Act) [29]. 

### 2.3. Questionnaire 

The survey was designed based on several validated questionnaires. 

Subscales from the Multidimensional Assessment of Interoceptive Awareness scale (MAIA) were used to evaluate the respondents’ self-reported interoceptive capabilities [30]. MAIA is an itemised state-trait questionnaire, making it possible via self-report to assess multiple dimensions of interoception, and it has been translated and validated in numerous languages. All versions are available online, including the Danish version used in the present study [31]. The subscales included in the study were: (1) Noticing: awareness of uncomfortable, comfortable, and neutral body sensations; (2) Attention Regulation: ability to sustain and control attention to body sensations; (3) Emotional Awareness: awareness of the connection between body sensations and emotional states; (4) Body Listening: active listening to the body for insight [30]. Version 2 (MAIA-2) was created in 2018 and includes improved psychometrics [31]. However, as a Danish-validated version is only available in Version 1, this version was used in the present study. The present study used a 5-point Likert reply scale with the anchor points ‘0’ = ‘Never’ to ‘4’ = ‘Always’. The final score from the subscales ranges from 1 to 5, where a higher score indicates higher awareness. For the Danish version, see [31].

The Snaith-Hamilton Pleasure Scale (SHAPS) [32] was used to determine the respondents overall self-reported ability to feel pleasure. The SHAPS is a 14-item self-assessment scale estimating the degree to which a person can experience pleasure or anticipate a pleasurable event (i.e., the hedonic tone or its absence, anhedonia). The scale covers four different domains of hedonic experiences: (1) food/drink, (2) sensory experiences, (3) social interaction and (4) pastimes/interests [32]. The questionnaire is built upon a 4-point ordinal Likert scale: ‘1’ = ‘Definitely disagree’, ‘2’ = ‘Disagree’, ‘3’ = ‘Agree’, and ‘4’ = ‘Definitely agree’. The final score ranges from 0 to 14. A score of 2 or less is interpreted as a ‘normal score’, meaning a normal ability to experience pleasure. A score of 3 or more is interpreted as an ‘abnormal score’, indicating an abnormal ability to experience pleasure. Thus, a higher total score indicates higher levels of anhedonia [32,33]. Moreover, this scale has been translated into several languages and validated as a precise and reliable measure of anhedonia state [32,33,34]. 

The subscale F2: Reliance on Hunger and Satiety Cues (RHSC) from the Intuitive Eating Scale (IES) was used to assess the respondents’ trust in their hunger and satiety sensations [35]. The IES contains three different subscales, giving a total of 23 items rated on a 5-point Likert scale with the anchors ‘1’ = ‘Strongly disagree’ to ‘5’ = ‘Strongly agree’. The final scores from the subscale range from 1 to 5, where a higher score indicates a higher level of intuitive eating. The IES was developed to measure subjective self-reports of intuitive eating and has been translated and validated into several languages, including Danish, which is the version used in the present study [35,36].

Further, a series of questions was developed specifically for the purpose of this study (Table S1). First, the respondents were asked to assess their relative intake frequency of different healthy and unhealthy snack foods by recalling the last week in terms of how often they ate different types of snack foods. The snack categories were chosen based on the works and definitions of the Danish National Food Institute and De Cock and colleagues (2017) [37,38,39]. The snack categories included a series of unhealthy and healthy snacks familiar to Danish consumers, used and pre-described in previous studies [37,38,39]. The unhealthy snack categories included: chocolate, cakes, sweets, chips, biscuits, ice cream, salted nuts and seeds, white bread, snack bars, crackers, desserts, milk-based products with a high fat/sugar content, and sugar-free candy. The healthy snack categories included: fruit, vegetables, wholemeal bread, milk-based products with a low fat/sugar content, unsalted nuts and seeds, dried fruit, and plant-based alternatives. 

Furthermore, the respondents were to choose different drivers for eating healthy and unhealthy snack foods via a check-all-that-apply scale. The drivers focused on energy and hunger sensations, hedonic aspects, habits, and cognitive, emotional, social, and cultural factors. The questions were inspired by articles by Cleobury and Tapper, (2014), Hess et al. (2016), and Verhoeven et al. (2015) [26,27,40].

The respondents were likewise asked to assess different types of post-ingestive sensations (PISs) as they were perceived after eating healthy and unhealthy snack foods. The post-ingestive sensations included appetite, wellbeing, and energy sensations and were rated on a 100 mm Visual Analog Scale (VAS) anchored by 0 = ‘I don’t feel it at all’ and 10 = ‘I feel it to an extremely high degree’ at the extreme ends. The questions were developed with inspiration from Duerlund and colleagues (2019a, 2019b) [8,9].

A modified version of the Food Pleasure Scale (FPS) was used to investigate the pleasurable aspects linked to snack food consumption [11]. The FPS is a self-report tool to measure quantitative and qualitative aspects of pleasure during food-related experiences and was used in the present study to determine the aspects driving the intake of healthy and unhealthy snack foods. The respondents were asked to rate the extent to which 20 different items contributed to their experiences of pleasure with healthy and unhealthy snacks, respectively. The items were rated on a 100 mm VAS anchored with 0 = ‘Not at all’, and 10 = ‘To an extremely high degree’. The copyright authors approved the scale modifications to make the questions as easy to understand and answer as possible. For example, one of the original questions was “I find pleasure in the taste of the food”, and this was modified for the purpose of the current study into “I find pleasure in the taste of healthy snack foods” and “I find pleasure in the taste of unhealthy snack foods”, respectively. 

As a guide for the respondents, definitions of healthy and unhealthy snack foods were included in the questionnaire when answering questions related to drivers, post-ingestive sensations (PISs), and food pleasure (FPS). The definitions were provided by The Danish National Food Institute [37]. Finally, sociodemographic and lifestyle variables were evaluated, including: gender, education level, weight, height, weight changes and smoking habits. 

### 2.4. Statistical Analyses 

First, descriptive statistics were applied to provide a description of the characteristics of the total group of respondents (n = 286), and further, the characteristics of respondents within the group of normal weight, overweight, and obese groups, separately. These weight groups were created based on the respondents’ BMI levels. BMI was calculated based on height and weight: weight (kg)/height (m)^2^. A total of n = 130 (45.5%) were included in the normal weight group, n = 80 (28%) were included in the ‘overweight’ group and n = 76 (26.5%) were included in the obese group. 

Additionally, all statistical analyses were applied to test: (1) differences between response variables within the total group of respondents n = 286 (dependent variables), and (2) differences between the three BMI groups (independent variables) for each response variable.

Using the Shapiro-Wilk test, the data were checked for normal distribution, and subsequent statistical tests were chosen accordingly. 

The Wilcoxon signed-rank test was utilised for the numerical data (i.e., FPS scores) to test for significant differences within dependent variables, whereas a McNemar test was used on categorical data (i.e., drivers for snacking). Furthermore, mixed-model ANOVA was used on the dependent variables to check for item differences (i.e., MAIA).

The Kruskal–Wallis Test was utilised for detecting significant differences in numerical values (i.e., age, MAIA, IES, FPS, and PISs) when the respondents were divided into three groups (independent variables), whereas chi^2^ tests were used for the categorical variables (i.e., gender, BMI, smoking, weight gain/loss, snack frequency intake, and drivers of snacking). In cases of theoretical counts lower than five, the data were double-checked with Fisher’s exact test, since this is considered a more appropriate test for smaller datasets. Dunn’s test was applied as a post hoc test in the event that Kruskal–Wallis results showed significant differences. All data were calculated using Microsoft Excel^®^ (Microsoft, 2305, Washington, DC, USA) and XLSTAT^®^ (Addison, 2023.1.2, New York, NY, USA) with the significance level set at α ≤ 0.05.

## 3. Results

### 3.1. Sociodemographic and Lifestyle Characteristics of the Three BMI Groups

The three groups proved to be somewhat different, as significant differences were found in terms of their age (*p* < 0.0001), gender (*p* = 0.001), education level (*p* = 0.001), and weight loss incidence (*p* = 0.004). Characteristics of the three BMI groups can be seen in Table 1. Regarding age, the group of normal weight respondents was significantly younger than the overweight and obese respondents. The overweight group contained significantly fewer females and more males compared to the group of normal weight (Fisher’s exact test: *p*-value = 0.010) and obese group (Fisher’s exact test: *p*-value = 0.010). No significant difference between the group of normal weight and obese was obtained.

Regarding the highest education level, the overweight group reported having a vocational education as their highest level of education significantly more than the normal weight group (Fisher’s exact test: *p*-value = 0.004). Significantly more overweight people had a secondary/high school education when compared to the obese group. Moreover, more of the normal weight had a secondary/high school education compared to the obese group (Fisher’s exact test: *p*-value = 0.001). There were significantly fewer people in the normal weight group that reported having a vocational education (Fisher’s exact test: *p*-value = 0.001) or short higher education as their highest education level (Fisher’s exact test: *p*-value = 0.017) compared to the obese group.

The normal weight group reported to be significantly more weight stable, as they reported fewer cases of weight loss of five or more kilos within the last six months (*p* = 0.040) compared to the two other groups. No significant difference were detected in terms of smoking.

### 3.2. Interoceptive Capabilities including Pleasure Sensations

**Figure 1:**
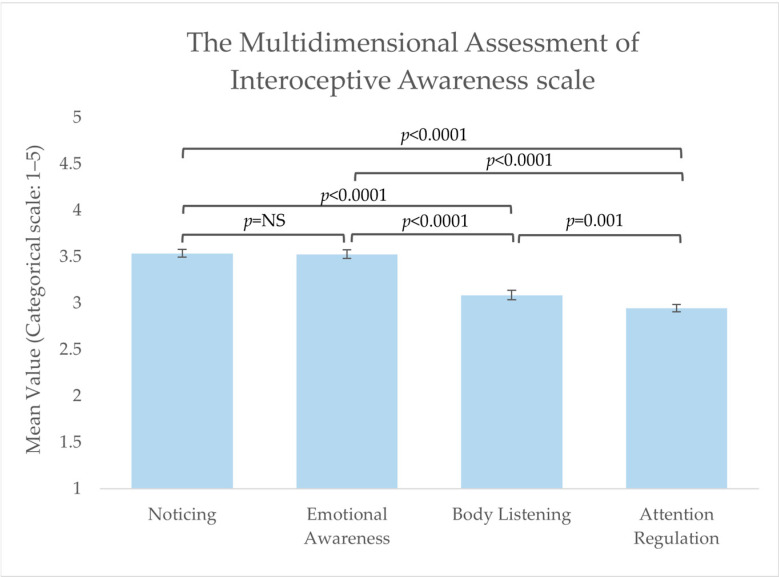
Mean score values from subscales of the Multidimensional Assessment of Interoceptive Awareness (MAIA) scale. Significant differences were obtained between the four subscales. NS: non-significant.

Four subscales from the MAIA scale [30] were used to investigate interoceptive abilities among the respondents, i.e., the subscales ‘Noticing’, ‘Attention Regulation’, ‘Emotional Awareness’, and ‘Body Listening’. Mean values for the total group of respondents on these subscales were: ‘Noticing’ = 3.5 (±0.68), ‘Attention Regulation’ = 2.9 (±0.69), ‘Emotional Awareness’ = 3.5 (±0.75), and ‘Body Listening’ = 3.1 (±0.86), indicating normal interoceptive capabilities among the respondents. Respondents scored significantly lower on ‘Attention Regulation’ compared to the other three subscales and significantly higher on ‘Noticing’ and ‘Emotional Awareness’ (see Figure 1 for significance levels). No significant differences were found between the three BMI groups on any of the subscales (no visualisation of these results is available).

The SHAPS scale was used to determine the respondents’ general ability to experience pleasure (i.e., not specifically food-related) [32]. For the total group of respondents, the majority showed a normal ability to feel pleasure (86.7%). No significant differences were found between the three BMI groups, implying no link between anhedonia and BMI in the current study (no visualisation of these results is available). 

### 3.3. Intuitive Eating

**Figure 2:**
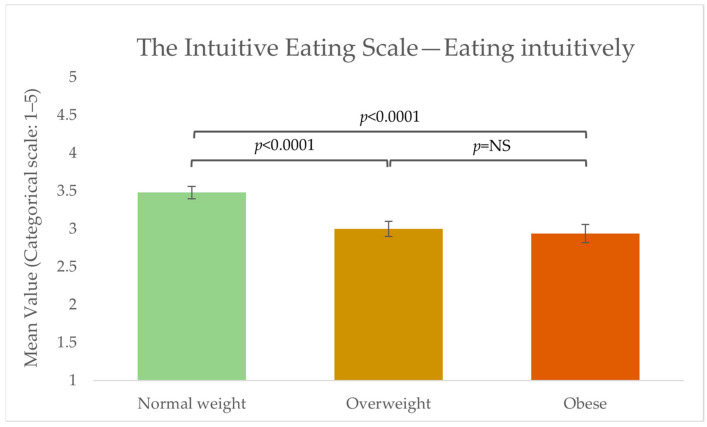
Mean score values from the Intuitive Eating Scale (IES) subscale ‘reliance on internal hunger and satiety cues’ from the three groups: normal weight (n = 130), overweight (n = 80), and obese (n = 76). Significant differences were obtained between the normal weight group and the overweight and obese groups. NS: non-significant.

To investigate intuitive eating, the respondents were asked to answer the reliance on internal hunger and satiety cues’ subscales from the IES [35]. The mean score across the total group of respondents was 3.2 (±0.98), thus slightly above the middle value, indicating a tendency to eat intuitively. Significant differences were found between the BMI groups, with the group of normal weight respondents being significantly more intuitive in their eating than the overweight and obese group (see Figure 2). 

### 3.4. Frequency of Snack Food Consumption

**Figure 3:**
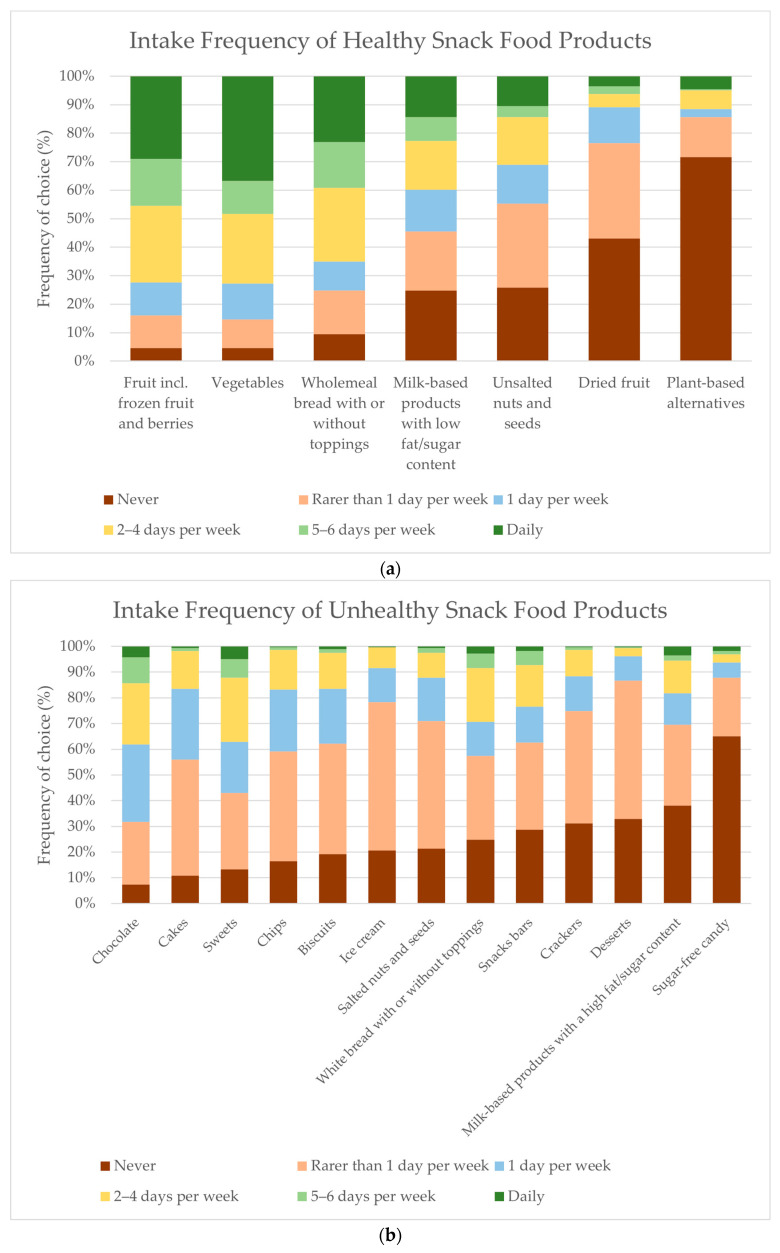
(**a**) Intake frequency of healthy snack products consumed within the last week as reported by all respondents (n = 286). (**b**) Intake frequency of unhealthy snack products consumed within the last week as reported by all respondents (n = 286).

To acquire insight into the frequency of their snack consumption, including healthy and unhealthy snack foods, the respondents were asked how often they ate typical snack products in the week before the study. For the total group of respondents concerning healthy snack food consumption, the top three snack food categories most frequently indicated to be eaten daily were: ‘vegetables’ (36.7%), ‘fruit’ (29%) and, ‘whole-meal bread’ (23.1%). the top three snack food categories most frequently indicated never to be eaten were: ‘plant-based alternatives’ (71.7%), ‘dried fruit’ (43%), and ‘unsalted nuts and seeds’ (25.9%) (see Figure 3a). According to the frequency intake of unhealthy snack foods for the total group of respondents the top three snack food categories most frequently indicated to be eatenwere:: ‘sweets’ (4.9%), ‘chocolate’ 4.2%), and ‘milk-based products with a high fat/sugar content’ (3.5%). The top three snack food categories most frequently indicated never to be eaten were: ‘sugar-free candy’ (65%), ‘milk-based products with a high fat/sugar content’ (38.1%), and ‘desserts’ (32.9%) (see Figure 3b). Overall, no differences were found between the three BMI groups in terms of the frequency of their intake of snack products, except for the intake frequency of ‘dessert’, where the Fisher’s exact test showed that the group of normal weight respondents reported eating desserts significantly more often than the group of overweight and obese respondents (*p* = 0.040, results not visualised). 

### 3.5. Drivers of Snack Food Consumption

**Figure 4:**
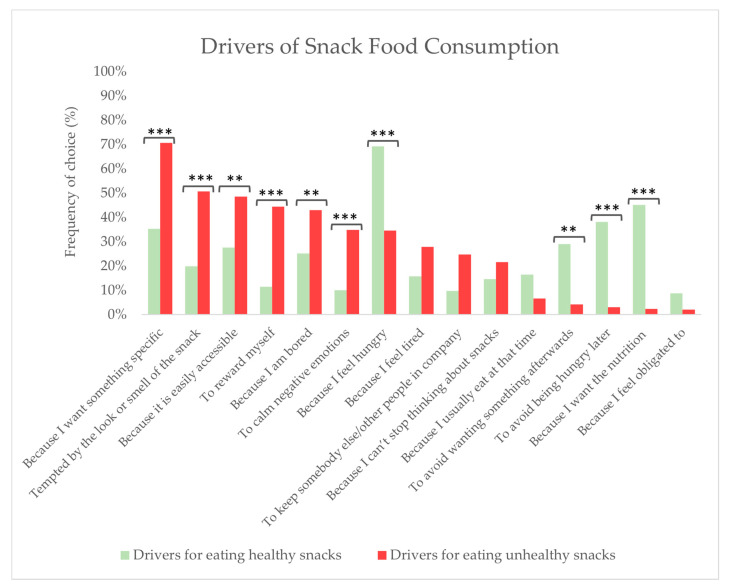
Drivers of snack food consumption for healthy and unhealthy snacks among all respondents (n = 286). Stars indicate a level of significance when comparing the drivers for consuming healthy snack foods with those for unhealthy snack foods: ** = *p* < 0.01, *** = *p* < 0.001.

Drivers for eating healthy and unhealthy snack foods were studied via check-all-that-apply scales. For the total group, the following reasons for eating healthy snack foods over unhealthy snack foods were reported significantly more often: the feeling of hunger (*p* < 0.0001), to avoid being hungry later (*p* < 0.0001), to prevent wanting something afterwards (*p* = 0.001), and because of the desire for nutrition (*p* < 0.0001) (see Figure 4). Conversely, eating unhealthy snack foods over healthy snack foods was significantly more often due to these reasons: the temptation of the appearance or smell of the snack (*p* < 0.0001), because it was easily accessible (*p* = 0.002), as a reward (*p* < 0.0001), because of boredom (*p* = 0.010) and to calm negative emotions (*p* = 0.001). 

When comparing the three BMI groups according to the drivers for eating healthy snacks, the three most often-occurring drivers were: for the normal weight group: the feeling of hunger (73.1%), the desire for nutrition (54.6%), and to avoid being hungry later (42.3%); for the overweight group: the feeling of hunger (62.5%), the desire for nutrition (36.3%), and wanting something specific (36.3%); and for the obese group: the feeling of hunger (69.7%), the desire for nutrition (38.2%), and to avoid being hungry later (38.2%). The three least frequent drivers were: for the normal weight group: because of the feeling of obligation (6.2%)’, to calm negative emotions (7.7%), and to keep somebody else in company (9.2%); for the overweight group: because of the feeling of obligation (7.5%), usually eat at that time (8.8%), and as a reward (10%); and for the obese group: to keep somebody else in company (9.2%), to calm negative emotions (10.5%), and as a reward’ (13.2%). Significantly more from the normal weight group reported eating healthy snack foods due to the desire for nutrition compared to the groups of overweight and obese respondents (*p* = 0.013, not visualised).

According to the drivers for eating unhealthy snack foods, the three most often reported drivers were: for the normal weight group: wanting something specific (73.8%), as a reward (53.8%), and the temptation of the appearance or smell of the food (50.8%); for the overweight group: wanting something specific (63.8%), because it is easily accessible (51.3%), and the temptation of the appearance or smell of the food (47.5%); and for the obese group: wanting something specific (72.4%), the temptation of the appearance or smell of the food (53.9%), and because it is easily accessible (51.3%). The three least frequently reported drivers were: for the normal weight group: because of a feeling of obligation (2.3%), to avoid being hungry later (3.1%), and because of the desire for nutrition (3.1%); for the overweight group: to avoid being hungry later (1.3%), ‘because of the desire for nutrition (1.3%), and to avoid wanting something afterwards (3.8%), and for the obese group: because of a feeling of obligations (0%), to avoid wanting something afterwards (2.6%), and because of the desire for nutrition (2.6%). Significantly more from the normal weight group reported eating unhealthy snacks due to rewarding themselves (*p* = 0.005). Conversely, significant differences were obtained between the normal weight group and obese group concerning eating to calm negative emotions (*p* = 0.007); in the group of obese respondents, significantly more individuals reported eating unhealthy snacks to calm negative emotions (results not visualised). 

### 3.6. Post-Ingestive Sensations Caused by Snack Food Consumption

**Figure 5:**
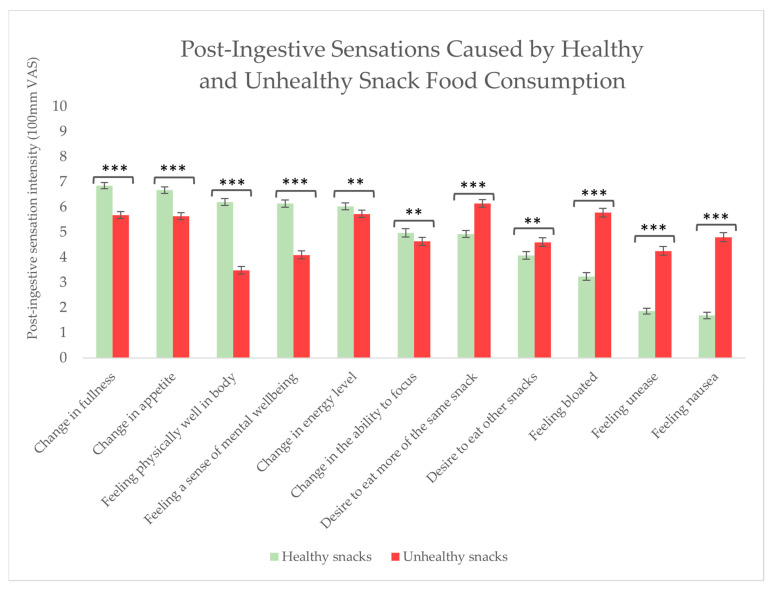
Post-ingestive sensations felt after eating healthy and unhealthy snacks among all respondents (n = 286). Stars indicate a level of significance when comparing the post-ingestive sensations experienced after eating healthy snack food with those experienced after eating unhealthy snack food: ** = *p* < 0.01, *** = *p* < 0.001.

The respondents were asked to assess the intensity of different internal bodily sensations after consuming healthy and unhealthy snack foods, respectively. For the total group of respondents, after eating healthy snacks, numerous post-ingestive sensations were felt to a significantly higher degree than after eating unhealthy snacks, including: a change in fullness (*p* < 0.0001), a change in appetite (*p* < 0.0001), feeling physical well (*p* < 0.0001), feeling mentally well (*p* < 0.0001), a change in energy level (*p* = 0.015), and a change in the ability to focus (*p* = 0.007). On the other hand, after eating unhealthy snacks, when compared to healthy snacks, respondents felt to a significantly higher degree: a desire to eat more of the same (*p* < 0.0001), a desire to eat other foods (*p* = 0.002), a bloated feeling (*p* < 0.0001), an uneasy feeling (*p* < 0.0001), or a nauseous feeling (*p* < 0.0001) (see Figure 5). 

When comparing the three BMI levels, no significant differences were found in the intensity of the post-ingestive sensations, neither after eating healthy nor after eating unhealthy snack foods (no visualisation available). 

### 3.7. Pleasurable Aspects of Healthy and Unhealthy Snack Foods

**Figure 6:**
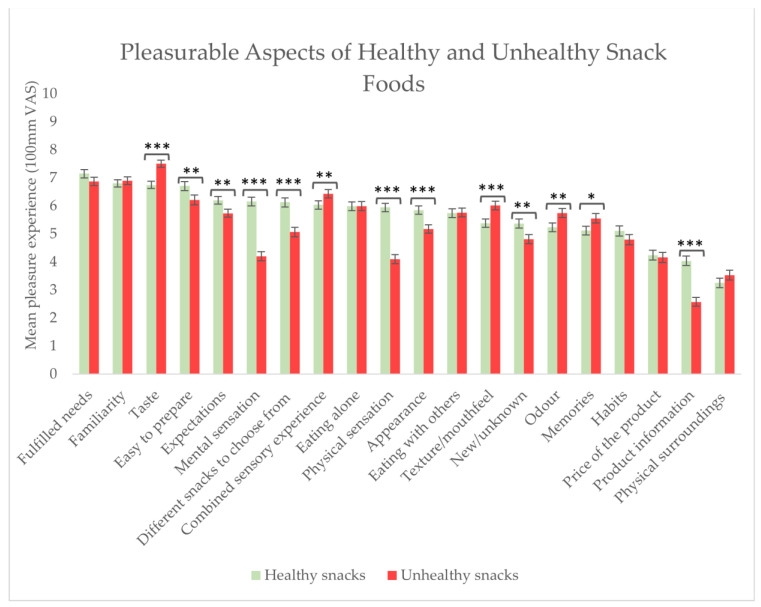
Food-related aspects that contributed to pleasure when eating healthy and unhealthy snacks reported among all respondents (n =286). Stars indicate a level of significant differences when comparing the importance of that aspect of pleasure when consuming healthy snack foods with its importance for unhealthy snack foods: * = *p* ≤ 0.05, ** = *p* < 0.01, *** = *p* < 0.001.

Looking more specifically into the pleasurable experience of eating snack foods, the respondents were asked to assess the extent to which different aspects of healthy and unhealthy snack food consumption brought pleasure. Aspects bringing pleasure to a significantly higher extent when eating healthy snack foods over unhealthy snack foods included: easy to prepare (*p* = 0.005), they confirmed expectations (*p* = 0.025), the mental sensations experienced (*p* < 0.0001), having different snacks to choose from (*p* < 0.0001), the physical sensations experienced (*p* < 0.0001), the appearance (*p* < 0.0001), new/unknown snacks (*p* = 0.003), and the product information (*p* < 0.0001) (see Figure 6). Conversely, aspects bringing pleasure to a significantly higher extent when eating unhealthy snack foods over healthy snack foods included: the taste (*p* < 0.0001), the combined sensory experience (*p* = 0.003), the texture/mouthfeel (*p* < 0.0001), the odour (*p* = 0.006), and the memories of previous snacks eaten (*p* = 0.025) (see Figure 6).

Several differences were found when comparing the three BMI groups according to aspects bringing pleasure, both in relation to healthy and unhealthy snack foods. 

**Figure 7:**
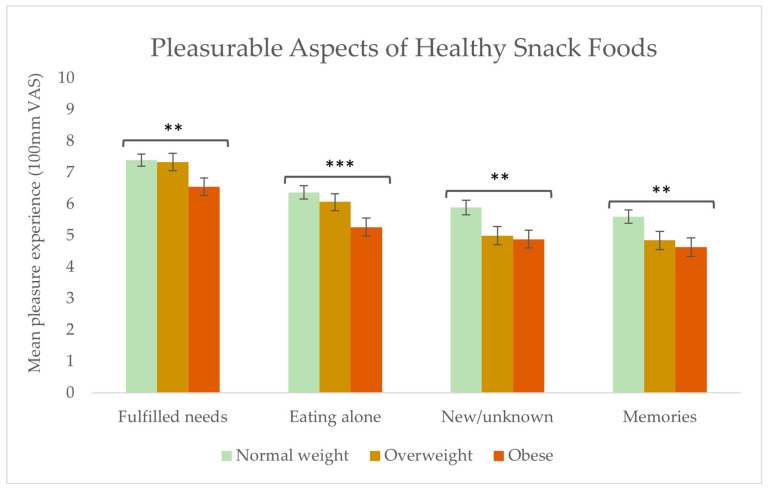
Food-related aspects contributing to pleasure when eating healthy snacks among the three groups: normal weight (n = 130), overweight (n = 80), and obese (n = 76). Stars indicate a level of significance when comparing the importance of that aspect of pleasure between the three BMI groups in relation to consuming healthy snack foods:, ** = *p* < 0.01, *** = *p* < 0.001.

Within the context of eating healthy snacks, significant differences were found in the pleasure experiences of the variables: fulfilled a need (*p* = 0.003), eating when alone (*p* = 0.001), eating new/unknown snacks (*p* = 0.002), and memories of previously eaten snacks (*p* = 0.012) (see Figure 7). For the normal weight and overweight groups, compared to the obese group, fulfilling a need and eating alone contributed more to a pleasurable experience with healthy snacks. For the groups of overweight and obese respondents, compared to the normal weight group, eating new/unknown snacks and memories of previously eaten snacks brought less pleasure with healthy snack foods. 

**Figure 8:**
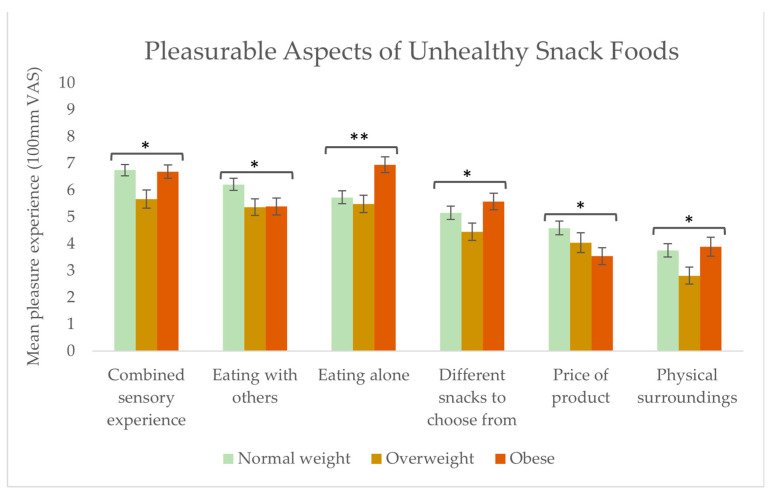
Food-related aspects contributing to pleasure when eating unhealthy snacks among the three groups: normal weight (n = 130), overweight (n = 80), and obese (n = 76). Stars indicate a level of significance when comparing the importance of that aspect of pleasure between the three BMI groups in relation to consuming unhealthy snack foods: * = *p* ≤ 0.05, ** = *p* < 0.01.

Within the context of eating unhealthy snack foods, significant differences were found in the pleasure experience of the variables: combined sensory experience (*p* = 0.050), eating with others (*p* = 0.019), eating alone (*p* = 0.002), different snacks to choose from (*p* = 0.030), price of the product (*p* = 0.033), and physical surroundings (*p* = 0.037) (see Figure 8). For the normal weight and obese groups, compared to the overweight group, the combined sensory experience and physical surroundings variables contributed to a higher degree to the pleasurable experience. For the group of normal weight respondents, compared to the groups of overweight and obese individuals, eating with others was more pleasurable. Conversely, the obese group found it more pleasurable to eat alone than the two other groups. Regarding the aspect of having different snacks to choose from, the obese group found significantly more pleasure to have different unhealthy snacks to choose from than the overweight group. Furthermore, the obese group found the price of the product to be significantly less important for bringing pleasure compared to the normal weight group.

## 4. Discussion

This study aimed to provide a detailed understanding of the interoceptive experience related to healthy and unhealthy snack food consumption among consumers from different BMI groups. Firstly, the study checked the respondents’ interoceptive capabilities and ability to feel pleasure and investigated their intuitive eating behaviours, snack food consumption frequency, the drivers of their snack food consumption, the aspects driving their feelings of pleasure, and the post-ingestive sensations linked to snack food consumption. Secondly, as previous research has suggested a potential difference in interoceptive experiences between BMI levels, this study was conducted on consumers from different BMI groups to study if the healthy and unhealthy snack food experiences varied between these groups. 

### 4.1. Interoceptive Capabilities and Intuitive Eating 

Interoceptive capabilities have previously been shown to differ among subjects from different BMI groups [17,41]. Robison et al. (2021b) concluded in a cross-sectional study that interoception deficits were associated with higher BMI. However, the causality remained unclear as to whether deficits in interoception contributed to or were a consequence of weight gain and obesity [17]. The present study detected no difference in self-report interoceptive capabilities between the three BMI groups. This indicates that the respondents had an equal self-report belief regarding their interoceptive capabilities regardless of their BMI levels (no visualization available).

In the present study, four subscales were used to describe aspects of the respondents’ general interoceptive awareness. Differences between the four subscales were found. Overall, the respondents were found: to be more aware of uncomfortable, comfortable, and neutral body sensations (Noticing), to be more aware of the connection between their body sensations and emotional states (Emotional Awareness), to have a lower ability to sustain and control their attention towards body sensations (Attention Regulation), and to listen actively to their body for insight (Body Listening). The results indicate that the respondents were characterised as being more aware of their uncomfortable, comfortable, and neutral body sensations (Noticing) and the connection between their body sensations and emotional states (Emotional Awareness) when compared to having the ability to sustain and control attention towards body sensations (Attention Regulation) and actively listen to the body for insight (Body Listening). Robinson and colleagues conducted a study (2021b) where they likewise studied the association between BMI and interoceptive capabilities [17]. In agreement with our study, they found no difference between BMI groups in the overall attention to interoceptive signals (i.e., the degree to which interoceptive signals are the object of attention [42]). However, when Robinson and colleagues asked subjects to pay attention to the signals, poorer self-reported interoceptive accuracy (i.e., the degree to which one’s interoceptive perception accurately represents the true state of the body [42]) was found among the higher BMI groups. Our results can be explained by the methodological focus of the MAIA scale, which measures self-report overall interoceptive awareness (also referred to as interoceptive sensibility)but not accuracy, specifically after an eating situation. Thus, it can be hypothesised that a scale measuring general self-report interoceptive awareness, though addressing different elements related to interoception, might not translate directly into interoceptive capabilities following an eating situation. Future studies conducted within the food domain are needed to clarify a potential association and causality between BMI and interoceptive capabilities, e.g., where subjects are trained or asked to pay attention to their interoceptive signals. The suggested hypothesis is supported by the results from the reliance on internal hunger and satiety cues’ subscale from the Intuitive Eating Scale [35]. The results showed significant differences between the group of normal weight compared to the groups of overweight and obese, indicating higher levels of intuitive eating among the group of normal weight respondents. Moreover, the normal weight group was found to be significantly more weight-stable than the overweight and obese subjects. This aligns with previous studies concluding that intuitive eating is connected with lower BMI, less body weight concerns and dieting, and better interoceptive capabilities [19,43,44]. Overall, the results across studies also indicate that attending to internal signals alone is not protective against body weight gain unless one can perceive those internal signals accurately [41]. Thus, a connection between poorer self-reported interoceptive accuracy and higher BMI has been proposed, whereas other studies have failed to find this association [45,46].

### 4.2. Snack Food Consumption and Drivers of Snack Intake

No difference in snack intake frequency was found between the three BMI groups. Several reasons can account for these results. The four most likely are: 1. the results of the present study depended on retrospection, where inaccurate memory could have biased the results; 2. a self-report bias, where respondents misreported their true snack intake to better fit their own self-perception [47]; 3. the results reflected a true picture where the snack intake frequency intake over the week did not vary between the BMI groups; and 4. the sensitivity of the questionnaire. One of the principles of intuitive eating is that nothing is forbidden and that every food can be eaten daily [35]. As the group of normal weight people did show a more-intuitive eating style, it is possible that the three groups ate the snack foods at a similar frequency; however, the intake quantity (amount) was not measured and could have potentially varied between BMI groups. However, the results may reflect the true intake. The categories included in the reply scale were potentially not detailed enough to show actual differences (e.g., no intake quantities were measured, only frequency). Further, the questionnaire only focused on snacks as an in-between meal, and thus no main meals or drinks were included in the questionnaire, which could have provided other results.

The respondents were asked to assess their drivers’ for eating healthy and unhealthy snack foods, respectively, to obtain a deeper insight into the drivers of snack intake. The results from the total group of respondents (n = 286) showed significant differences in relation to the drivers for choosing the two types of snack food categories. Healthy snack foods were most often chosen for reasons related to internal bodily sensations: due to the feeling of hunger, to avoid being hungry later, because of the want for certain nutrition, and also as a strategy for avoiding being hungry later. Conversely, the reasons for eating unhealthy snacks were often due to hedonic factors, e.g., being tempted by the look or smell of the food or because of wanting something specific, and cognitive factors/emotions, e.g., because of boredom, as a reward, or as a way to calm negative emotions. The results thereby suggest that healthy and unhealthy snacks are chosen to fulfil different needs. Our results are in line with previous studies investigating drivers for eating snacks, where hunger tends to be associated with the consumption of health-promoting foods, and snacking in the absence of hunger can lead to the consumption of fats, sugars, and sodium-rich foods, as seen in [35].

Differences were also found between the three BMI groupsin relation to drivers for eating healthy and unhealthy snack foods. The normal weight group more often chose healthy snacks because of their nutritional relevance. These results can potentially be explained by the fact that lower BMI and intuitive eating are linked to better interoceptive abilities, which can influence decision-making towards healthier food choices [48]. Unhealthy snacks were more often chosen for the reasons of calming negative emotions by the obese BMI group, and the normal weight group ate unhealthy snacks more often for the reasons of rewarding themselves. Thus, it can be argued that snack food consumption in the context of obesity is linked to a coping strategy where foods can calm negative emotions and reflect a more emotional eating style. Emotional eating is defined as the urge or tendency to eat regardless of hunger when facing negative emotions [49], and emotional eating has, in previous studies, been connected to higher BMI levels e.g., in [50], and lower interoception [51]. Therefore, the higher tendency to eat unhealthy snacks due to calming negative emotions could be a reflection of emotional eating and a lower interoceptive ability among the obese subjects, in combination with the fact that unhealthy snacks, have been proven to be able to bring comfort [52].

### 4.3. Post-Ingestive Sensations after Snack Food Consumption

The intensity of the respondents’ post-ingestive sensations after snack food consumption was rated to obtain detailed insight into the sensations felt after consumption of healthy and unhealthy snack foods. For the total group of respondents (n = 286), healthy snack food consumption (compared to unhealthy snack consumption) was to a higher extent associated with: feeling a change in fullness and appetite, feeling physically and mentally well, and a change in energy level and ability to focus. Conversely, unhealthy snack food consumption was to a higher extent linked with a desire to eat more of the same food as well as other foods, feeling bloated, feeling unease, and nausea. These results show that eating unhealthy snacks results in unpleasant sensations yet a desire to eat more. In contrast, the post-ingestive sensations following consumption of healthy snacks concluded in pleasant sensations. The results suggest sensory desires to be stronger and overrule negative sensations felt as a consequence of eating unhealthy snacks. This result is in line with previous research on a related topic: Attuquayefio and colleagues (2016) conducted a study investigating the effect of a high-fat, high-sugar diet on wanting levels and concluded that a high-fat, high-sweet dietary intake is associated with poor inhibition of food-related wanting when satiated. Further, they concluded that the desire to consume unhealthy food is less affected by physiological state [53]. 

No association between post-ingestive sensations and BMI levels could be detected, indicating that post-ingestive sensations are similar regardless of BMI levels. 

### 4.4. Pleasurable Aspects of Healthy and Unhealthy Snack Foods

The respondents’ ability to perceive pleasure was measured via the SHAPS scale [32]. The majority of the respondents had a normal ability to experience pleasure, and no differences were found between the three BMI groups. Using the SHAPS allowed us to investigate the overall self-report conscious ability to experience pleasure among the respondents, in contrast to studies investigating reward via fMRI e.g., [54,55]. While several studies from this discipline indicated different food-related reward responses between different BMI groups, e.g., [54,55], our particular study did not detect differences in the overall self-report conscious ability to experience pleasure when using the self-report SHAPS between the three BMI groups. As this could be a sign of no difference, we need to consider that the SHAPS scale measures the ability to feel overall pleasure by including four aspects that normally induce the feeling of pleasure and wellbeing. The scale is used in clinical contexts to measure anhedonia and is not designed to measure pleasure in food-related contexts exclusively [32]. Therefore, studying the food-related aspects of pleasure further was also relevant, as we did by including a modified version of the FPS questionnaire [11]. 

Despite finding no differences in the overall self-report conscious ability to experience pleasure (measured by SHAPS) between BMI levels, the study did find differences in different food-related aspects bringing pleasure in relation to healthy and unhealthy snack food consumption, both among the total group of respondents (n = 286) and between the three BMI groups. Differences were seen among the total group of respondents in relation to which aspects brought pleasure, when eating healthy snacks compared to eating unhealthy snacks. When eating unhealthy snacks compared to healthy snacks, aspects contributing to a more pleasurable experience included: the sensory experience, i.e., the taste, the texture/mouthfeel, the odour and the feeling of combined sensory experience. On the other hand, aspects like: easy to prepare, confirmed pleasure expectations, mental sensations, physical sensations, different snacks to choose from, appearance, new/unknown snacks, and product information contributed to more pleasurable experiences with healthy snacks compared to unhealthy snack consumption. Thus, these results suggest that the pleasure gained from unhealthy snacks is due to the intrinsic product characteristics, whereas the pleasure gained from healthy snack food consumption is due to the healthiness factor and the feeling of doing something good for the body physically and mentally. Our results align with previous results indicating that the information given about a food product’s healthiness can affect perceptions of the product. For example, in a series of four experiments, Raghunathan and colleagues (2006) found that when information about the healthiness of foods is provided, the less healthy the food is presented to the respondent, the better its inferred taste, the more it is enjoyed during actual consumption, and the greater the preference for it in choice tasks when a hedonic goal is more (versus less) salient [56]. 

The three BMI groups gave different importance ratings to the aspects driving pleasure with healthy snack foods. The group of normal weight individuals experienced more pleasure (than the overweight and obese groups) when healthy snacks fulfilled a need or were new/unknown, and when they were consumedalone. Further, the memory of eating healthy snack foods was to a higher extent linked with a pleasurable experience than in the overweight and obese groups. In the context of unhealthy snack consumption, the group of normal weight respondents found social company significantly more pleasurable than the overweight and obese groups. Conversely, the obese group found it significantly more pleasurable to eat unhealthy snacks alone. These results are likely related to the shame and stigmatisation previously reported by overweight and obese people when eating unhealthy snacks [57]. Further, these results can be explained by the results of our investigation into the drivers of unhealthy snack food consumption, where the obese group more often chose to eat unhealthy snacks as a way to calm negative emotions. In contrast, the normal weight group chose to eat unhealthy snacks as a reward. The mental states promoting eating in the two situations are very different: negatively driven by obesity/overweight and positively driven by normal weight. Further, having different snacks to choose from was significantly more pleasurable among the obese group. These results align with previous studies, concluding that obese subjects tend to consume a greater variety of energy-dense foods than normal weight subjects, as seen in [58,59], and our results showing the importance of the combined sensory experience, which requires a broad sensory flavour stimulation to be achieved.

Studying the aspects bringing pleasure with healthy and unhealthy snack food consumption can expand our understanding of the drivers of healthy and unhealthy snack consumption overall and also among different consumers, including across different BMI groups.

### 4.5. Strengths and Limitations of the Current Study 

To our knowledge, this is the first time that the subjective experience of snack food consumption has been studied with this level of detail. This study has revealed novel insights into interoceptive sensations involved in snack food consumption, which can be beneficial in developing sustainable long-term strategies for dietary behaviour changes. Future research is encouraged to broaden the conceptualisation of interoception from largely being appetite-focused to incorporating pleasure-drivers in order to better understand human food and eating behaviours.

In relation to the results based on the MAIA scale and the four subscales used in the present study, it should be considered that these results can be a consequence of the methodology approach, as the MAIA scale includes only a few items for assessing interoception [60], combined with not using the full questionnaire in the present study. Further studies using this approach are recommended to use the full length of the questionnaire or consider combining the MAIA scale with other suitable measures. 

The current study relies on self-report reporting’s, which are highly beneficial when studying subjective experiences, as was carried out in the present study. However, our results should also be interpreted cautiously, especially regarding snack intake frequency, as the results depend on retrospection, and memory can be inaccurate. Further, the well-known self-report bias of respondents could cause them to tweak their answers for a better fit with their self-perception [47]. Therefore, there is a possibility that the reported intake frequency of snack food consumption does not reflect actual consumption, and differences in snack frequency could potentially be found if studied using other methods. Moreover, based on the self-report nature of the study, the BMI categorisation might not be completely accurate. The researchers calculated the BMI of each participant based on the information about weight and height that they provided. While acknowledging the risk of inaccuracy among the self-reported weight and height data, the current approach was applied to avoid respondents reporting their own BMI levels directly. With this current approach, some variance in respondents’ self-reported weight and height levels can be allowed without changing the BMI grouping of the respondent.

Finally, in this study, the majority of the respondents were women (84.2%), and differences were seen between the three BMI groups according to gender, age, education level, and weight stability. This study does not address gender-, age-, or education level-related differences, as the current study focused on BMI levels, and neither can this study be regarded as representative of the Danish population. Still, it cannot be ruled out that gender and age differences could contribute to the results. However, no previous research indicates that this should be the case. 

## 5. Conclusions

This study aimed to explore subjective interoceptive experience of snack food consumption in detail. This was conducted via an online self-report questionnaire that included an investigation of the respondents’ interoceptive capabilities and ability to feel pleasure, intuitive eating behaviour, snack food consumption frequency, the drivers of snack food consumption, pleasurable aspects linked to healthy and unhealthy snack food consumption, and the post-ingestive sensations experienced. The study was further conducted on consumers from different BMI segments to contribute to the pool of evidence of potential differences between these groups. 

The study showed normal self-reported interoceptive and pleasure perception capabilities across the sample, with no differences between BMI levels. Yet, a more intuitive eating style was found for the group holding normal BMI compared to overweight and obese respondents. No difference between the BMI groups was found in snack food intake frequency. However, differences in drivers for snack food consumption were found between the consumption of healthy and unhealthy snack foods, indicating healthy snacks to be consumed based on internal bodily sensations, i.e., hunger sensations and conversely, unhealthy snacks to be consumed based on the hedonic characteristics of the snack food and to emotional factors felt by the consumer. Drivers for snack food consumption were found to vary between BMI groups, indicating higher BMI to be associated with eating unhealthy snacks for emotional reasons. 

The study found differences in post-ingestive sensations felt after consumption of healthy and unhealthy snacks. Eating unhealthy snacks resulted in unpleasant sensations, yet a desire to eat more, and in contrast, post-ingestive sensations followed by healthy snack consumption concluded in pleasant sensations. No difference was found between BMI levels in their post-ingestive sensations. 

Furthermore, the study found differences in thedegree to which different aspects brought pleasure with healthy and unhealthy snack foods. Our results suggest that the pleasure gained from unhealthy snacks is due to the intrinsic product characteristics, whereas pleasure gained from healthy snack food consumption is due to the healthiness factor and the feeling of doing something good for the body physically and mentally. Finally, differences were found between BMI levels and aspects driving pleasure in relation to healthy and unhealthy snack food consumption. The group of normal weight experienced more pleasure (than the overweight and obese group) when healthy snacks fulfilled a need, were new/unknown, and were consumed alone. Further, the memory of eating healthy snack foods was to a higher extent linked with a pleasurable experience than in the group of overweight and obese. In the context of unhealthy snack consumption, the group of normal weight found social company significantly more pleasurable than the group of overweight and obese. Conversely, the group of obese found it significantly more pleasurable to eat unhealthy snacks alone. Further, having different snacks to choose from was significantly more pleasurable among the obese group. 

Taken together, the study provides detailed and novel insight into subjectively experienced drivers for healthy and unhealthy snack food consumption and the sensations linked to this consumption. From a scientific perspective, this study provides a basis of knowledge on the sensations felt in relation to snack food consumption, which is useful for further research of interoception as a strategy for dietary control. From an applicable perspective, this study highlights different motivations for engaging in healthy versus unhealthy snack food consumption, which can be useful when developing strategies for dietary counselling.

## Figures and Tables

**Table 1:** Characteristics of all respondents, divided into three BMI groups.

Sample Characteristics	Total	Normal Weight	Overweight	Obese	*p*-Values
Number of respondents	286 (100%)	130 (45.5%)	80 (28%)	76 (26.5%)	α ≤ 0.05
Gender					*p* = 0.001
Female	240 (84.2%)	117 (90%)	56 (70.9%)	67 (88.2%)	
Male	45 (15.8%)	13 (10%)	23 (29.9%)	9 (11.8%)	
Age *	37.94 (±14.54)	34.23 (± 13.23)	40.82 (±15.62)	41.26 (±13.86)	*p* < 0.0001
BMI ^1,^*	27.24 (±6.40)	22.14 (±1.89)	27.26 (±1.35)	35.95 (±5.08)	*p* < 0.0001
Highest completed educational level					*p* = 0.001
Primary school/elementary school education	16 (5.6%)	6 (4.6%)	3 (3.9%)	7 (9.2%)	
Secondary/high school education	34 (11.9%)	22 (16.9%)	10 (12.7%)	2 (2.6%)	
Vocational education	32 (11.2%)	5 (3.8%)	13 (16.5%)	14 (18.4%)	
Short higher education (2 years)	19 (6.7%)	4 (3.1%)	6 (7.6%)	9 (11.8%)	
Medium-length higher education (3–4 years)	101 (35.4%)	50 (38.5%)	26 (32.9%)	25 (32.9%)	
Long higher education (5+ years)	83 (29.1%)	43 (33.1%)	21 (26.6%)	19 (25%)	
Smoking status					NS
Frequent smoker	27 (9.5%)	9 (6.9%)	8 (10.1%)	10 (13.2%)	
Occasional smoker	225 (78.9)	107 (82.3%)	57 (72.2%)	61 (80.3%)	
Non-smoker	33 (11.6%)	14 (10.8%)	14 (17.7%)	5 (6.6%)	
Weight gain (5+ kg within the last six months)					NS
Weight not stable	78 (27.3%)	27 (20.8%)	26 (32.5%)	25 (32.9%)	
Weight stable	208 (72.7%)	103 (79.2%)	54 (67.5%)	51 (67.1%)	
Weight loss (5+ kg within the last six months)					*p* = 0.004
Weight not stable	90 (31.5%)	28 (21.5%)	32 (40%)	30 (39.5%)	
Weight stable	196 (68.5%)	102 (78.5%)	48 (60%)	46 (60.5%)	

* Mean ± standard deviation (range), ^1^ BMI: Body Mass Index, NS: non-significant.

## Data Availability

Data is contained within the article or supplementary material.

## Supplementary Materials

The following supporting information can be downloaded at https://www.mdpi.com/article/10.3390/foods13030438/s1: Table S1. Overview of questionnaire phrasing and response variables used in the study.
