# Supplementary material for: Sensing the Snacking Experience: Bodily Sensations Linked to the Consumption of Healthy and Unhealthy Snack Foods—A Comparison between Body Mass Index Levels
Source: Foods. 2024 Jan 29;13(3):438. doi: 10.3390/foods13030438 (PMC10855362; doi:10.3390/foods13030438)
Supplement: Supplementary file 1 [file foods-13-00438-s001.zip › Table S1 Questionnaire.pdf]

Supplementary Material

Table S1. Overview of questionnaire phrasing and response variables used in the study.

Category / Response variable	Question in Danish	Question in English	Danish reply scale	English reply scale
Screening questions	Hvad er din alder?	What is your age?	Angiv venligst din alder i hele tal	Please indicate your age in whole numbers
Screening questions	Lider du af allergi, sygdom eller tager du medicin, som udelukker visse fødevarer fra din kost og/eller påvirker din appetit?	Do you suffer from allergies or illness, or do you take medication that excludes certain foods from your diet and/or affects your appetite?	• Ja<br>• Nej	• Yes<br>• No
**Snack frequency questions**				
Introduction text	Udfyld venligst i nedenstående tabel, hvor ofte du spiser følgende fødevarer som mellemmåltid. Tænk på den seneste uge.<br><br>Et mellemmåltid er defineret af DTU Fødevareinstituttet som enhver mad, der indtages mellem eller efter de tre hovedmåltider (morgenmad/brunch, frokost og aftensmad).<br><br>Vi er ikke interesseret i mængden der spises (da det kan variere), blot i hvor ofte du spiser følgende mellemmåltider:	Please fill in the table below for how often you eat the following foods as a snack. Think about the latest week.<br><br>A snack is defined as any food not consumed in a main meal, either between or after meals (breakfast/brunch, lunch and dinner).<br><br>We are not interested in the amount (and know it can vary), only in how often you eat the following snacks:	• Aldrig<br>• Sjældnere end 1 dag om ugen<br>• 1 dag om ugen<br>• 2-4 dage om ugen<br>• 5-6 dage om ugen<br>• Dagligt	• Never<br>• Rarer than 1 day per week<br>• 1 day per week<br>• 2-4 days per week<br>• 5-6 days per week<br>• Daily
	Slik (f.eks. lakrids, vingummi, bolcher, skumslik, tyggegummi)	Sweets (e.g. liquorice, wine gum candies, marshmallows, chewing gum)		
	Sukkerfrit slik	Sugar-free candy		

	Chokolade (f.eks. chokoladebar, flødeboller, marcipan/konfekt, chokoladepålæg)	Chocolate (e.g. chocolate bar, cream buns, marzipan/confectionery, chocolate spread)
	Kager (f.eks. gærkage, formkage/skærekage, wienerbrød, tørkage, trøfler/romkugler, flødeskumskager, pandekager, tærte)	Cakes (e.g. cakes, pastry, truffles/rum balls, whipped cream cakes, pancakes, pie)
	Småkager (f.eks., cookies, chokolade- eller karamelkiks, andre søde kiks)	Biscuits (e.g. chocolate, caramel biscuits, other sweet biscuits)
	Kiks (f.eks. Saltholdige kiks, saltkiks, kiks med smag)	Crackers (e.g. saltine crackers, flavoured crackers)
	Snackbars (f.eks. müslibar, energibar, proteinbar, mælkesnitte)	Snack bars (e.g. muesli bar, energy bar, protein bar, milk bars)
	Is (f.eks. flødeis/mælkeis, sodavandsis inkl. Veganske is)	Ice cream (e.g. ice cream, ice lollies, vegan ice creams)
	Desserter (f.eks. chokolademousse, fromage, budding)	Desserts (e.g. chocolate mousse, pudding)
	Chips (f.eks. Chips, flæskesvær, popcorn)	Chips (e.g. Chips, pork crackle, popcorn)
	Tørret frugt (f.eks. rosiner, abrikoser)	Dried fruits and vegetables, including processed and preserved fruit and vegetables (e.g. raisins, apricots, instant dried vegetables, canned fruit, candied dates, plum candy)
	Frugt, inkl. frossen frugt og bær (f.eks. æble, banan, pære, blåbær)	Fresh fruit (e.g. apple, banana)
	Grøntsager (f.eks. gulerod, agurk, snack peber)	Vegetables (e.g. vegetables sticks)
	Nødder og frø uden salt (f.eks., solsikkefrø, valnødder, mandler)	Unsalted nuts and seeds, (e.g. sunflower seeds, walnuts, almonds)
	Nødder og frø med salt (f.eks. peanuts)	Salted nuts and seeds (e.g. peanuts)

	Mælkebaseret produkter med højt indhold af fedt/sukker (f.eks. frugtyoghurt, ostehaps etc.)	Milk-based products with a high fat/sugar content (e.g. fruit yoghurt, cheese curds, etc.)		
	Mælkebaseret produkter med lavt indhold af fedt/sukker (f.eks. yoghurt naturel, skyr, mager ostesnacks)	Milk-based products with low fat/sugar content (e.g. natural yoghurt, low-fat cheese snacks)		
	Plantebaserede alternativer (f.eks. cremet havre, cremet soja)	Plant-based alternatives (e.g. creamed oats, creamed soya)		
	Lyst brød med eller uden pålæg (f.eks. hvedeboller, lyst toastbrød)	White bread with or without toppings (e.g. wheat buns, white toast bread)		
	Fuldkornsbrød med eller uden pålæg (f.eks. rugbrød, fuldkornsboller- eller toastbrød)	Wholemeal bread with or without toppings (e.g. rye bread, wholemeal-buns or toast bread)		
	Andre mellemmåltider ikke nævnt ovenfor, venglist angiv	Other snacks not mentioned above, please specify		
**Drivers for eating healthy snacks**				
	Hvad er årsagen til at du spiser SUNDE MELLEMMÅLTIDER? Vælg venligst alle de svarkategorier, der passer på dig.<br><br>Sunde mellemmåltider er defineret af DTU Fødevareinstituttet, som fødevare der har en højere næringsstoftæthed (højt indhold af kostfibre og mikronæringsstoffer, som vitaminer, mineraler), og en lavere energitæthed, f.eks. grøntsagsstave, frugt, fuldkornsprodukter (rugbrød).<br><br>Jeg spiser et sundt mellemmåltid…	What is the reason you eat HEALTHY SNACKS? Please select all the answer categories that apply to you.<br><br>Healthy snacks are defined by the DTU Food Institute as food that has a higher nutrient density (high content of dietary fibre and micronutrients, such as vitamins and minerals) and a lower energy density, for example, vegetable sticks, fruit, whole grain products (rye bread).<br><br>I eat a healthy snack…	Vælg venligst alle de svarkategorier, der passer på dig	Please select all the answer categories that apply to you
Internal	Fordi jeg føler mig sulten	Because I feel hungry		

Internal	For at undgå at blive sulten senere	To avoid being hungry later		
Hedonic eating	Fordi jeg bliver fristet af madens udseende eller duft	Because I am tempted by the look or smell of the food		
Hedonic eating	Fordi jeg ikke kan stoppe med at tænke på mad	Because I can't stop thinking about food		
Hedonic eating	Fordi jeg har lyst til noget bestemt	Because I want something specific		
Hedonic eating	For at undgå lyst til noget efterfølgende	To avoid wanting something afterwards		
Emotional eating	Fordi jeg føler mig træt	Because I feel tired		
Emotional eating	Fordi jeg keder mig	Because I am bored		
Emotional eating	For at dulme negative følelser (f.eks. stress, tristhed, anspændthed)	To calm negative emotions (e.g. stress, sadness, tension)		
Emotional eating	For at belønne mig selv (f.eks. hvis jeg har arbejdet hårdt, har en god dag, har det godt eller er i godt humør)	To reward myself (e.g. I worked hard, having a good day, feeling good, or are in a good mood)		
Social/food culture environment	For at holde en anden/andre med selskab	To keep somebody else/other people with company		
Social/food culture environment	Fordi jeg føler mig forpligtet til det	Because I feel obligated to		
Social/food culture environment	Fordi det er nemt tilgængeligt	Because it is easily accessible		
Habit	Fordi jeg plejer at spise på det tidspunkt	Because I usually eat at that time		
Other	Fordi jeg ønsker næring (f.eks. vitaminerne, energien, kostfibrene)	Because I want the nutrition (e.g. the vitamins, the energy, the dietary fibre)		
**Drivers for eating unhealthy snacks**				
	Hvad er årsagen til at du spiser USUNDE MELLEMMÅLTIDER? Vælg venligst alle de svarkategorier, der passer på dig.<br>Usunde mellemmåltider er defineret	What is the reason you eat UNHEALTHY SNACKS? Please select all the answer categories that apply to you.<br><br>Unhealthy snacks are defined by the DTU	Vælg venligst alle de svarkategorier, der passer på dig	Please select all the answer categories that apply to you

	af DTU Fødevareinstituttet, som næringsfattige og energitætte fødevarer, som normalt har et højt fedt-, salt- og/eller sukker, og energiindhold og et lavt næringsstofindhold (lavt indhold af kostfibre og mikronæringsstoffer, som vitaminer, mineraler), som f.eks., slik, chokolade, kage, chips – inklusiv sukkerfrie produkter.<br>Jeg spiser et usundt mellemmåltid…	Food Institute as nutrient-poor and energy-dense foods, which usually have a high fat, salt and/or sugar, and energy content and a low nutrient content (low content of dietary fibre and micronutrients, such as vitamins, minerals) for example, sweets, chocolate, cake, chips – including sugar-free products.<br>I eat an unhealthy snack…
Internal	Fordi jeg føler mig sulten	Because I feel hungry
Internal	For at undgå at blive sulten senere	To avoid being hungry later
Hedonic eating	Fordi jeg bliver fristet af madens udseende eller duft	Because I am tempted by the look or smell of the food
Hedonic eating	Fordi jeg ikke kan stoppe med at tænke på mad	Because I can't stop thinking about food
Hedonic eating	Fordi jeg har lyst til noget bestemt	Because I want something specific
Hedonic eating	For at undgå lyst til noget efterfølgende	To avoid wanting something afterwards
Emotional eating	Fordi jeg føler mig træt	Because I feel tired
Emotional eating	Fordi jeg keder mig	Because I am bored
Emotional eating	For at dulme negative følelser (f.eks. stress, tristhed, anspændthed)	To calm negative emotions (e.g. stress, sadness, tension)
Emotional eating	For at belønne mig selv (f.eks. hvis jeg har arbejdet hårdt, har en god dag, har det godt eller er i godt humør)	To reward myself (e.g. I worked hard, having a good day, feeling good, or are in a good mood)
Social/food culture environment	For at holde en anden/andre med selskab	To keep somebody else/other people with company
Social/food culture environment	Fordi jeg føler mig forpligtet til det	Because I feel obligated to

Social/food culture environment	Fordi det er nemt tilgængeligt	Because it is easily accessible		
Habit	Fordi jeg plejer at spise på det tidspunkt	Because I usually eat at that time		
Other	Fordi jeg ønsker næring (f.eks. vitaminerne, energien, kostfibrene)	Because I want the nutrition (e.g. the vitamins, the energy, the dietary fibre)		
**The modified version of The Food Pleasure Scale (FPS) – healthy snacks**				
Introduction text	Spørgsmålene i dette skema drejer sig om nydelse ved fødevarer. Du bedes venligst vurdere, i hvor høj grad du finder nydelse fra visse aspekter omkring indtag af sunde mellemmåltider. Tænk på, hvordan du har det lige nu og lav en markering på skalaen.<br><br>Sunde mellemmåltider er defineret af DTU Fødevareinstituttet, som fødevare der har en højere næringsstoftæthed (højt indhold af kostfibre og mikronæringsstoffer, som vitaminer, mineraler), og en lavere energitæthed, f.eks. grøntsagsstave, frugt, fuldkornsprodukter (rugbrød).<br><br>I hvor høj grad bidrager følgende aspekter omkring SUNDE MELLEMMÅLTIDER til fødevare-nydelse?	The following questions are about pleasure in relation to food. Please rate to what extent you find pleasure from certain aspects of eating healthy snacks. Think about how you feel right now and make a mark on the scale.<br><br>Healthy snacks are defined by the DTU Food Institute as food that has a higher nutrient density (high content of dietary fibre and micronutrients, such as vitamins and minerals) and a lower energy density, for example, vegetable sticks, fruit, whole grain products (rye bread).<br><br>To what extent do the following aspects of HEALTHY SNACKS contribute to food pleasure?	100 mm VAS skala: Overhovedet ikke - i ekstrem høj grad	100 mm VAS scale: Not at all - to an extremely high degree
Memories	Når jeg tænker tilbage på situationer hvor jeg indtog sunde mellemmåltider så er dette minde forbundet med nydelse	When I think back at a situation where I eat healthy snacks, this memory is pleasurable		

Expectations	Når jeg tænker tilbage på situationer hvor jeg havde lyst til - og indtog - sunde mellemmåltider, så fik jeg indfriet mine forventninger til hvor nydelsesfulde disse fødevarer var	When I think back at a situation where I desired to eat healthy snacks, my expectations around enjoyment were confirmed
Fulfilled needs	Når jeg indtager sunde mellemmåltider, så er det ofte fordi de skal opfylde et behov jeg har (fx give energi, tilfredsstille en craving, gøre mig mæt)	When I eat healthy snacks, I want the foods to fulfil a need (e.g. bring energy, satisfy a craving, make me feel full)
Different snacks to choose from	Når jeg indtager sunde mellemmåltider, så finder jeg nydelse i at have flere forskellige sunde mellemmåltider at vælge imellem	When I eat healthy snacks, I find pleasure in having more options to choose from
Habits	Når jeg indtager sunde mellemmåltider, så er det ofte vanebetonet (fx på samme tidspunkt om dagen, i de samme situationer)	When I eat healthy snacks, it is often a habitual behaviour (e.g. same time of day, same situations)
Product information	Når jeg køber sunde mellemmåltider, så er den information jeg får omkring produktet vigtig for, at jeg finder nydelse ved fødevaren (fx information om produktionsmetode, indhold af ingredienser)	When I buy healthy snacks, the information about the product is important for me to feel pleasure around the food (e.g. information about production method, content of ingredients)
Physical surroundings	Når jeg indtager sunde mellemmåltider, så er de fysiske omgivelser vigtige for, at jeg finder nydelse ved fødevaren	When I eat healthy snacks, the physical surroundings are important for me to feel pleasure around the food
Familiarity	Jeg finder nydelse i at spise sunde mellemmåltider, som jeg kender godt	I find pleasure in eating healthy snacks that are familiar to me
New/unknown	Jeg finder nydelse i at spise nye/ukendt sunde mellemmåltider	I find pleasure in eating healthy snacks that are novel to me

Appearance	Jeg finder nydelse i måden sunde mellemmåltider ser ud på	I find pleasure in the appearance of healthy snacks
Odour	Jeg finder nydelse i duften af sunde mellemmåltider	I find pleasure in the smell of healthy snacks
Taste	Jeg finder nydelse i smagen af sunde mellemmåltider	I find pleasure in the taste of healthy snacks
Texture/mouthfeel	Jeg finder nydelse i måden sunde mellemmåltider føles i munden	I find pleasure in the texture of healthy snacks
Combined sensory experience	Når jeg indtager sunde mellemmåltider, er det den samlede sensoriske oplevelse (udseende, duft, smag og konsistens) som tilfredsstiller mig	When I eat healthy snacks, it is the combined sensory experience (appearance, smell, taste and texture) that satisfies me
Physical sensation	Når jeg har indtaget sunde mellemmåltider, så har jeg en nydelsesfuld fysisk fornemmelse i kroppen efterfølgende	When I have eaten healthy snacks, I feel pleasurable physical sensations in the body
Mental sensation	Når jeg har indtaget sunde mellemmåltider, så har jeg en nydelsesfuld mental fornemmelse efterfølgende	When I have eaten healthy snacks, I feel pleasurable mental sensations
Eating alone	Jeg nyder at indtage sunde mellemmåltider, når jeg er alene	I find pleasure in eating healthy snacks when I am alone
Eating with others	Jeg nyder at indtage sunde mellemmåltider, når jeg er sammen med andre	I find pleasure in eating healthy snacks when I am with others
Easy to prepare	Når jeg vælger sunde mellemmåltider, er det vigtig at de er lette at tilberede (eller ikke kræver tilberedning) for at jeg finder nydelse	When I choose healthy snacks, it is important that they are easy to prepare (or do not require any preparation) in order for me to feel pleasure
Price of product	Når jeg køber sunde mellemmåltider, så er prisen vigtig for, at jeg finder nydelse ved fødevaren	When I buy healthy snacks, the price is important for me to feel pleasure around the food

The modified version of The Food Pleasure Scale (FPS) – unhealthy snacks				
Introduction text	Spørgsmålene i dette skema drejer sig om nydelse ved fødevarer. Du bedes venligst vurdere, i hvor høj grad du finder nydelse fra visse aspekter omkring indtag af usunde mellemmåltider. Tænk på, hvordan du har det lige nu og lav en markering på skalaen.<br><br>Usunde mellemmåltider er defineret af DTU Fødevareinstituttet, som næringsfattige og energitætte fødevarer, som normalt har et højt fedt-, salt- og/eller sukker, og energiindhold og et lavt næringsstofindhold (lavt indhold af kostfibre og mikronæringsstoffer, som vitaminer, mineraler), som f.eks., slik, chokolade, kage, chips – inklusiv sukkerfrie produkter.<br><br>I hvor høj grad bidrager følgende aspekter omkring USUNDE MELLEMMÅLTIDER til fødevare-nydelse?	The following questions are about pleasure in relation to food. Please rate to what extent you find pleasure from certain aspects of eating UNHEALTHY SNACKS. Think about how you feel right now and make a mark on the scale.<br><br>Unhealthy snacks are defined by the DTU Food Institute as nutrient-poor and energy-dense foods, which usually have a high fat, salt and/or sugar, and energy content and a low nutrient content (low content of dietary fibre and micronutrients, such as vitamins, minerals) for example, sweets, chocolate, cake, chips – including sugar-free products.<br><br>To what extent do the following aspects of UNHEALTHY SNACKS contribute to food pleasure?	100 mm VAS skala: Overhovedet ikke - i ekstrem høj grad	100 mm VAS scale: Not at all - to an extremely high degree
Memories	Når jeg tænker tilbage på situationer hvor jeg indtog usunde mellemmåltider så er dette minde forbundet med nydelse	When I think back at a situation where I eat unhealthy snacks, this memory is pleasurable		
Expectations	Når jeg tænker tilbage på situationer hvor jeg havde lyst til og indtog usunde mellemmåltider, så fik jeg	When I think back at a situation where I desired to eat unhealthy snacks, my		

	indfriet mine forventninger til hvor nydelsesfulde disse fødevarer var	expectations around enjoyment were confirmed
Fulfilled needs	Når jeg indtager usunde mellemmåltider, så er det ofte fordi de skal opfylde et behov jeg har (fx give energi, tilfredsstille en craving, gøre mig mæt)	When I eat unhealthy snacks, I want the foods to fulfil a need (e.g. bring energy, satisfy a craving, make me feel full)
Different snacks to choose from	Når jeg indtager usunde mellemmåltider, så finder jeg nydelse i at have flere forskellige usunde mellemmåltider at vælge imellem	When I eat unhealthy snacks, I find pleasure in having more options to choose from
Habits	Når jeg indtager usunde mellemmåltider, så er det ofte vanebetonet (fx på samme tidspunkt om dagen, i de samme situationer)	When I eat unhealthy snacks, it is often a habitual behaviour (e.g. same time of day, same situations)
Product information	Når jeg køber usunde mellemmåltider, så er den information jeg får omkring produktet vigtig for, at jeg finder nydelse ved fødevaren (fx information om produktionsmetode, indhold af ingredienser)	When I buy unhealthy snacks, the information about the product is important for me to feel pleasure around the food (e.g. information about production method, content of ingredients)
Physical surroundings	Når jeg indtager usunde mellemmåltider, så er de fysiske omgivelser vigtige for, at jeg finder nydelse ved fødevaren	When I eat unhealthy snacks, the physical surroundings are important for me to feel pleasure around the food
Familiarity	Jeg finder nydelse i at spise usunde mellemmåltider, som jeg kender godt	I find pleasure in eating unhealthy snacks that are familiar to me
New/unknown	Jeg finder nydelse i at spise nye/ukendt usunde mellemmåltider	I find pleasure in eating unhealthy snacks that are novel to me
Appearance	Jeg finder nydelse i måden usunde mellemmåltider ser ud på	I find pleasure in the appearance of unhealthy snacks

Odour	Jeg finder nydelse i duften af usunde mellemmåltider	I find pleasure in the smell of unhealthy snacks
Taste	Jeg finder nydelse i smagen fra usunde mellemmåltider	I find pleasure in the taste of unhealthy snacks
Texture/mouthfeel	Jeg finder nydelse i måden usunde mellemmåltider føles i munden	I find pleasure in the texture of unhealthy snacks
Combined sensory experience	Når jeg indtager usunde mellemmåltider, er det den samlede sensoriske oplevelse (udseende, duft, smag og konsistens) som tilfredsstiller mig	When I eat unhealthy snacks, it is the combined sensory experience (appearance, smell, taste and texture) that satisfies me
Physical sensation	Når jeg har indtaget usunde mellemmåltider, så har jeg en nydelsesfuld fysisk fornemmelse i kroppen efterfølgende	When I have eaten unhealthy snacks, I feel pleasurable physical sensations in the body
Mental sensation	Når jeg har indtaget usunde mellemmåltider, så har jeg en nydelsesfuld mental fornemmelse efterfølgende	When I have eaten unhealthy snacks, I feel pleasurable mental sensations
Eating alone	Jeg nyder at indtage usunde mellemmåltider, når jeg er alene	I find pleasure in eating unhealthy snacks when I am alone
Eating with others	Jeg nyder at indtage usunde mellemmåltider, når jeg er sammen med andre	I find pleasure in eating unhealthy snacks when I am with others
Easy to prepare	Når jeg vælger usunde mellemmåltider, er det vigtig at de er lette at tilberede (eller ikke kræver tilberedning) for at jeg finder nydelse	When I choose unhealthy snacks, it is important that they are easy to prepare (or do not require any preparation) in order for me to feel pleasure
Price of product	Når jeg køber usunde mellemmåltider, så er prisen vigtig for, at jeg finder nydelse ved fødevaren	When I buy unhealthy snacks, the price is important for me to feel pleasure around the food
**Post-ingestive sensation variables from eating healthy snacks**		

Introduction text	I dette skema kommer der nogle spørgsmål omkring i hvor høj grad du mærker forskellige indre kropslige fornemmelser i forbindelse med indtag af sunde mellemmåltider. Læs hvert udsagn og lav en markering på skalaen, hvordan det forholder sig i dit liv.<br><br>Sunde mellemmåltider er defineret af DTU Fødevareinstituttet, som fødevare der har en højere næringsstoftæthed (højt indhold af kostfibre og mikronæringsstoffer, som vitaminer, mineraler), og en lavere energitæthed, f.eks., grøntsagsstave, frugt, fuldkornsprodukter (rugbrød).<br><br>I hvor høj grad mærker du forskellige indre kropslige fornemmelser når du indtager SUNDE MELLEMMÅLTIDER?	The following questions are the extent to which you feel various internal bodily sensations in connection with the consumption of healthy snacks. Read each statement and mark on the scale how it relates to your life.<br><br>Healthy snacks are defined by the DTU Food Institute as food that has a higher nutrient density (high content of dietary fibre and micronutrients, such as vitamins, minerals) and a lower energy density, for example, vegetable sticks, fruit, whole grain products (rye bread).<br><br>To what extent do you feel different internal bodily sensations when you eat HEALTHY SNACKS?	100 mm VAS skala: Mærker jeg overhovedet ikke - mærker jeg i ekstrem høj grad	100 mm VAS scale: I don't feel it at all - I feel it to an extremely high degree
Appetit	Når jeg har indtaget sunde mellemmåltider, kan jeg mærke at min sult ændrer sig	When I have eaten healthy snacks, I can feel my appetite change		
Appetit	Når jeg har indtaget sunde mellemmåltider, kan jeg mærke jeg at min mæthed ændrer sig	When I have eaten healthy snacks, I can feel my fullness change		
Appetit	Når jeg har indtaget sunde mellemmåltider, kan jeg mærke uro i kroppen	When I have eaten healthy snacks, I can get a feeling of unease		
Appetit	Når jeg har indtaget sunde mellemmåltider, kan jeg blive oppustet	When I have eaten healthy snacks, I can feel bloated		

Appetit	Når jeg har indtaget sunde mellemmåltider, kan jeg få kvalme	When I have eaten healthy snacks, I can feel nausea		
Appetit	Når jeg har indtaget sunde mellemmåltider, får jeg lyst til at spise mere af det samme	When I have eaten healthy snacks, I feel a desire to eat more of the same foods		
Appetit	Når jeg har indtaget sunde mellemmåltider, får jeg lyst til at spise andre fødevarer	When I have eaten healthy snacks, I feel a desire to eat other foods		
Wellbeing	Når jeg har indtaget sunde mellemmåltider, mærker jeg en fornemmelse af mental velvære	When I have eaten healthy snacks, I feel a sense of mental well-being		
Wellbeing	Når jeg har indtaget sunde mellemmåltider, mærker jeg en fornemmelse af fysisk velvære i kroppen	When I have eaten healthy snacks, I feel physical well in my body		
Energy sensations	Når jeg har indtaget sunde mellemmåltider, kan jeg mærke at min koncentrationsevne ændrer sig	When I have eaten healthy snacks, I can feel my ability to focus level change		
Energy sensations	Når jeg har indtaget sunde mellemmåltider, kan jeg mærke at mit energiniveau ændrer sig	When I have eaten healthy snacks, I can feel my energy level change		
**Post-ingestive sensation variables from eating unhealthy snacks**				
Introduction text	I dette skema kommer der nogle spørgsmål omkring i hvor høj grad du mærker forskellige indre kropslige fornemmelser i forbindelse med indtag af usunde mellemmåltider. Læs hvert udsagn og lav en markering på skalaen, hvordan det forholder sig i dit liv.<br><br>Usunde mellemmåltider er defineret af DTU Fødevareinstituttet, som	The following questions are the extent to which you feel various internal bodily sensations in connection with the consumption of unhealthy snacks. Read each statement and mark on the scale how it relates to your life.<br><br>Unhealthy snacks are defined by the DTU Food Institute as nutrient-poor and energy-dense foods, which usually have a high fat, salt and/or sugar, and energy	100 mm VAS skala: Mærker jeg overhovedet ikke - mærker jeg i ekstrem høj grad	100 mm VAS scale: I don't feel it at all - I feel it to an extremely high degree

	næringsfattige og energitætte fødevarer, som normalt har et højt fedt-, salt- og/eller sukker, og energiindhold og et lavt næringsstofindhold (lavt indhold af kostfibre og mikronæringsstoffer, som vitaminer, mineraler), som f.eks., slik, chokolade, kage, chips – inklusiv sukkerfrie produkter.<br><br>I hvor høj grad mærker du forskellige indre kropslige fornemmelser når du indtager USUNDE MELLEMMÅLTIDER?	content and a low nutrient content (low content of dietary fibre and micronutrients, such as vitamins, minerals) for example, sweets, chocolate, cake, chips – including sugar-free products.<br><br>To what extent do you feel different internal bodily sensations when you eat UNHEALTHY SNACKS?
Appetit	Når jeg har indtaget usunde mellemmåltider, kan jeg mærke at min sult ændrer sig	When I have eaten unhealthy snacks, I can feel my appetite change
Appetit	Når jeg har indtaget usunde mellemmåltider, kan jeg mærke jeg at min mæthed ændrer sig	When I have eaten unhealthy snacks, I can feel my fullness change
Appetit	Når jeg har indtaget usunde mellemmåltider, kan jeg mærke uro i kroppen	When I have eaten unhealthy snacks, I can get a feeling of unease
Appetit	Når jeg har indtaget usunde mellemmåltider, kan jeg blive oppustet	When I have eaten unhealthy snacks, I can feel bloated
Appetit	Når jeg har indtaget usunde mellemmåltider, kan jeg få kvalme	When I have eaten unhealthy snacks, I can feel nausea
Appetit	Når jeg har indtaget usunde mellemmåltider, får jeg lyst til at spise mere af det samme	When I have eaten unhealthy snacks, I feel a desire to eat more of the same foods
Appetit	Når jeg har indtaget usunde mellemmåltider, får jeg lyst til at spise andre fødevarer	When I have eaten unhealthy snacks, I feel a desire to eat other foods

Wellbeing	Når jeg har indtaget usunde mellemmåltider, mærker jeg en fornemmelse af mental velvære	When I have eaten unhealthy snacks, I feel a sense of mental well-being		
Wellbeing	Når jeg har indtaget usunde mellemmåltider, mærker jeg en fornemmelse af fysisk velvære i kroppen	When I have eaten unhealthy snacks, I feel physical well in my body		
Energy sensations	Når jeg har indtaget usunde mellemmåltider, kan jeg mærke at min koncentrationsevne ændrer sig	When I have eaten unhealthy snacks, I can feel my ability to focus level change		
Energy sensations	Når jeg har indtaget usunde mellemmåltider, kan jeg mærke at mit energiniveau ændrer sig	When I have eaten unhealthy snacks, I can feel my energy level change		
**Lifestyle/sociodemographic questions**				
Weight	Hvad er din vægt?	What is your weight?	Angiv venligst i kg.	Please enter in kg.
Height	Hvad er din højde?	What is your height?	Angiv venligst i cm.	Please enter in cm.
Weight status	Har din vægt ændret sig 5+ kg indenfor det sidste halve år?	Has your weight changed 5+ kg within the last six months?	Vægtøgning: Ja/Nej<br>Vægttab: Ja/Nej	Weight gain: Yes/No<br>Weight loss: Yes/No
Gender	Hvad er dit biologiske køn ved fødsel?	What is your biological sex at birth?	• Kvinde<br>• Mand	• Female<br>• Male
Smoking status	Ryger du?	Do you smoke?	• Ja, ofte<br>• Ja, sjældent<br>• Nej	• Yes, often<br>• Yes, seldom<br>• No
Educational level	Hvad er dit højest afsluttede uddannelsesniveau?	What is your highest level of education?	• Folkeskole / grundskole<br>• Gymnasial uddannelse<br>• Erhvervsfaglig uddannelse	• Primary school/elementary school<br>• Secondary/ High-school education

		• Kort videregående uddannelse f.eks. erhvervsakademiuddannelse (2 år)<br>• Mellemlang videregående uddannelse (3-4 år)<br>• Lang videregående uddannelse (5+ år)	• Vocational education<br>• Short higher education (2 years)<br>• Medium higher education (3-4 years)<br>• Longer higher education (5+ years)
